# Usefulness of Cone-Beam CT-Based Liver Perfusion Mapping for Evaluating the Response of Hepatocellular Carcinoma to Conventional Transarterial Chemoembolization

**DOI:** 10.3390/jcm10040713

**Published:** 2021-02-11

**Authors:** Sun Young Choi, Kyung Ah Kim, Woosun Choi, Yohan Kwon, Soo Buem Cho

**Affiliations:** 1 Department of Radiology, School of Medicine, Ewha Womans University, Seoul 07985, Korea; kingnose80@gmail.com; 2 Department of Radiology, St. Vincent’s Hospital, College of Medicine, Suwon 16247, Gyeonggi-do, Korea; bellenina@daum.net; 3 Department of Radiology, Chung-ang University Hospital, Seoul 06973, Korea; radiochoi527@cau.ac.kr; 4 Department of Radiology, Ajou University Hospital, Suwon 16499, Gyeonggi-do, Korea; whitetsm@hanmail.net

**Keywords:** hepatocellular carcinoma, chemoembolization, cone-beam CT, perfusion imaging

## Abstract

We investigated the cone beam computed tomography (CBCT)-based-liver-perfusion-mapping usefulness during transarterial chemoembolization (TACE) in hepatocellular carcinoma (HCC) to access treatment response and predict outcomes. From October 2016 to September 2018, 42 patients with HCCs scheduled for conventional TACE were prospectively enrolled. Three reviewers evaluated the unenhanced and contrast-enhanced CBCT and CBCT-based-liver-perfusion-mapping of each tumor. Parenchymal blood volume (PBV) was measured. The operator’s judgment on the technical results was recorded. Response outcome was determined on follow-up CT or magnetic resonance imaging, according to the modified Response Evaluation Criteria in Solid Tumors. Diagnostic performance for detection of a viable tumor was evaluated using multiple logistic regression with C-statistics. CBCT-based-liver-perfusion-mapping and the maximum PBV of the tumor were significant in multiple logistic regression analysis of response (*p* < 0.0001, *p* = 0.0448, respectively), with C-statistics of 0.9540 and 0.7484, respectively. Diagnostic accuracy of operator’s judgment was 79.66% (95%CI 69.39%–89.93%). Diagnostic performance of CBCT-based-liver-perfusion-mapping showed a high concordance in three reviewers. The mean PBV of tumor, maximum PBV of tumor, and mean PBV of liver significantly decreased after TACE (each *p* < 0.001). In ROC curve analysis, the AUC for prediction of residual tumor by the maximum PBV of tumor after TACE was 0.7523, with 80.8% sensitivity and 60.6% specificity.

## 1. Introduction

Quantifying tumor angiogenesis is important for evaluating disease progression and monitoring response to therapy in hepatocellular carcinoma (HCC) [1,2]. This information can be obtained through tissue sampling, but it is not commonly used due to its invasive nature. Conventional computed tomography (CT) perfusion imaging is a noninvasive, quantitative technique that assesses tissue perfusion by measuring the passage of a bolus of iodinated contrast medium through a vascular system and allows calculation of several physiological parameters, including parenchymal blood volume (PBV) [3,4,5]. However, this approach cannot provide intraprocedural measures of tissue perfusion.

The objective treatment response to transarterial chemoembolization (TACE) was a surrogate marker of overall survival and thus early response to TACE is one of the predictors of outcome in HCC [6,7,8,9,10,11]. Therefore, the final goal of TACE should be the achievement of complete necrosis of the tumor, particularly of small tumors, at any time-point. Cone-Beam CT (CBCT) has become a key procedural step during TACE because of its ability to provide information relevant to lesion detection, catheter navigation, and assessment of technical success in three dimensions, in contrast to two dimensions in standard angiography [12,13]. However, CBCT techniques in conventional TACE with ethiodized oil have some limitations. Unenhanced CBCT is limited in accurately evaluating viable tumor portion because it can only estimate the ethiodized oil deposition in the tumor. In dual-phase CBCT, any residual viable tumor may also be missed due to the heterogeneity of ethiodized oil deposition throughout the tumor and the regional noncancerous hepatic parenchyma, particularly in small tumors, because of the similar density of ethiodized oil and iodine contrast agent, which can lead to incomplete TACE, worsening the prognosis [14,15].

With the development of CBCT technology, CBCT-based-perfusion-imaging has been shown to provide complementary information on tissue perfusion during the TACE procedure [16,17]. We previously conducted a retrospective study of the efficacy of CBCT-based-liver-perfusion-mapping during TACE and concluded that it has reliable diagnostic performance for evaluating treatment response by qualitative visual assessment, and for detection of any residual viable portion by quantitative perfusion analysis [16]. In the present study, we prospectively validated the usefulness of CBCT-based-liver-perfusion-imaging during TACE for qualitative treatment response assessment after conventional TACE, for quantitative perfusion assessment in treated HCC, and for assessing its usefulness as a surrogate marker of the early treatment response during TACE in order to optimize treatment for each patient.

## 2. Materials and Methods

### 2.1. Patient Selection and Study Design

This was a prospective, single-center study approved by the relevant institutional review board, obtaining written informed consent from each patient. From October 2016 to September 2018, 42 patients with HCC, confirmed on dynamic CT or magnetic resonance imaging (MRI), and scheduled for conventional TACE were enrolled. 

Patient eligibility criteria was followed; inclusion criteria: Age > 19 years with HCC unsuitable for resection or local ablation; Barcelona Clinic Liver Cancer stage A or B; HCC image diagnosis based on arterial enhancement and washout in the portal venous or delayed phase; Eastern Cooperative Oncology Group performance status 0 or 1; and preserved liver function (Child‒Pugh Class A or B); exclusion criteria: Another primary tumor; advanced liver disease (serum bilirubin level > 5 mg/dL, AST or ALT > 5 × the upper limit of normal or 250 U/L); advanced tumor disease (portal vein thrombosis or distant metastasis); contraindications for doxorubicin administration; renal insufficiency; alcohol abuse; or were pregnant or lactating. During this study period, only the first TACE procedure was analyzed in each enrolled patient. If breathing control failed or the patient did not cooperate during the procedure, CBCT images were not obtained during TACE for HCC and the patient was excluded (*n* = 3). Patients who did not undergo CT or MRI for follow-up after TACE were also excluded (*n* = 4). One case, who was confirmed pathologically through surgical resection after TACE rather than by CT or MRI, was included. Finally, 42 people were initially enrolled in this study, but 7 people dropped out and a total of 35 people were finally enrolled in this study (Table 1).

### 2.2. TACE Technique and Post-Procession of CBCT Data

All conventional TACE were performed by two radiologists (14 and 7 years of interventional experience) in the same flap panel angiographic system with post-processing software (Artis Q; Siemens Healthcare, Erlangen, Germany). Conventional TACE was performed as selectively as possible through the lobar, segmental, or subsegmental arteries, depending on the degree of malignancy and the underlying liver function. In conventional TACE, anticancer drug was a mixture of ethiodized oil (lipiodol; Andre Guerbet, Aulnay-sous-Bois, France) and doxorubicin hydrochloride (Adriamycin RDF; Ildong Pharmaceutical, Seoul, Korea); this mixture was administered into the feeding arteries through a 2.0-F microcatheter (Progreat Alpha; Terumo, Tokyo, Japan). The amount of ethiodized oil (lipiodol) was 4 to 10 mL, and the amount of doxorubicin was 10 to 50 mg. For embolization, 300–500 µm calibrated gelatin sponge particles (Cali-Gel; Alicon, Hangzhou, Zhejiang, China) were mixed with 10 mg doxorubicin hydrochloride (Adriamycin RDF) and contrast agent, were administered into the feeding arteries until the portal vein was visualized throughout the embolization area. Cessation of feeding flow was confirmed at least 5 min after verification of congestion of the feeding flow [16]. 

The CBCT parameters were 0.5° increment, 211° circular trajectory, 512 × 512 matrix in projections, and 48-cm field-of-view in 2D raw data. To obtain the CBCT scan, the acquisition protocol consisted of two rotations: An initial rotation (mask run) followed by injection of contrast medium, and then a second rotation (fill run) after an appropriate scan delay [16,18,19,20]. The CBCT scan was obtained both before and after chemoembolization.

**Figure 1:**
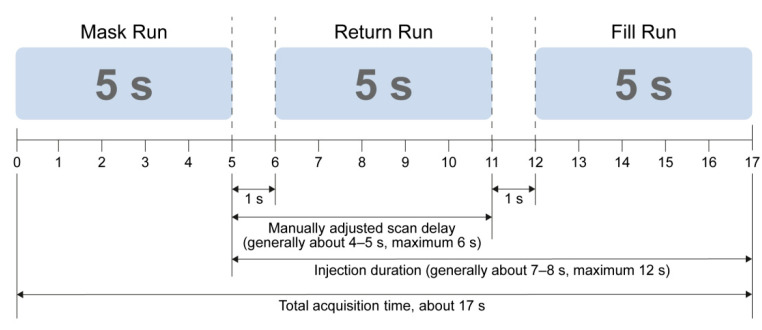
Schematic diagram of parenchymal blood volume (PBV) acquisition time.

A contrast free mask run took 5 s. The fill run was obtained under hepatic artery angiography by positioning a 5-F catheter tip at the proper hepatic artery with injection of 50% diluted iodine contrast agent in normal saline at a 3 mL/s injection rate for a duration of generally about 7–8 s, to a maximum 12 s. The scan delay in CBCT acquisition was manually adjusted according to the previously performed celiac artery angiography, with a range of 4–5 s, applied during the return run [16]. Overall, 17 s were required to obtain CBCT images (Figure 1). The acquired data were sent to a workstation (Syngo DynaPBV body; Siemens Healthcare, Erlangen, Germany). CBCT-based-perfusion-liver-images were obtained automatically from CBCT data using post-processing software. Operator opinions on the possibility of residual viable tumor after chemoembolization were recorded during the procedure.

### 2.3. Image Analysis

Three radiologists, with 4, 5, and 6 years of interventional experience, respectively, who did not participate in the TACE performed the analysis. They were blinded to the treatment outcome on follow-up radiological examinations or histological examination obtained after TACE. They reviewed and assessed the level of confidence visual scoring of viable tumors on each CBCT image on the mask run (L-CBCT), the CBCT images on the fill run (CE-CBCT), and CBCT-based-liver-perfusion-mapping images, obtained pre- and post-chemoembolization. Reviewers analyzed consecutive images obtained from each imaging modality for all patients in a single session. After completing the imaging analysis for 1 modality, they similarly reviewed the images for another modality. For image analysis, a 4-point scale was applied: 0, definitely no viable tumor; 1, likely no viable tumor; 2, possible viable tumors; and 3, definite viable tumors. A score of 0 or 1 was considered to represent absence of a viable tumor, and a score of 2 or 3 was taken to represent the presence of a tumor [16]. 

Depending on the imaging modality, the presence of residual viable tumor was determined as follows: The portion without ethiodized oil deposition within the treated tumor on L-CBCT, the contrast-enhancing portion within the treated tumor on CE-CBCT, and the presence of a nodular or mass-like increased perfusion area (excluding the expected vascular area) on CBCT-based-liver-perfusion-mapping. The region-of-interest (ROI) corresponding to each tumor, and the whole liver on an axial image containing the maximum diameter of the tumor was drawn on CBCT-based-perfusion-mapping images acquired on pre- and post-chemoembolization. 

The mean PBV (PBV_mean_) and the maximum PBV (PBV_max_) were obtained as perfusion parameters on pre- and post-chemoembolization. Perfusion parameter analysis was performed by the operator who performed the TACE. 

The modified Response Evaluation Criteria in Solid Tumors (mRECIST) guideline was used for the analysis of response to TACE on three types of CBCT images obtained after chemoembolization, and obtained at the first follow-up visit with dynamic CT or MRI. A direct comparison was made between the mRECIST response on each CBCT image obtained after chemoembolization and on the dynamic CT or MR images obtained at the first follow-up visit. 

A viable tumor suspected from each CBCT images obtained after chemoembolization was deemed a true residual tumor if a viable tumor was identified on follow-up dynamic liver CT or MRI at the corresponding site. If surgical resection was performed after TACE, the suspected viable tumor was confirmed by histological examination. The operator’s judgment as to whether the viable portion remaining on the CBCT-based-liver-perfusion-mapping obtained after chemoembolization was also analyzed.

### 2.4. Statistical Analysis

Interobserver agreement among the three reviewers about the treatment response of TACE for each tumor, with a binary outcome (negative decision for a viable tumor: Score of 0 or 1 vs. positive decision: score of 2 or 3), based on each three types of CBCT images, was measured by means of the kappa (k) coefficient. The strength of agreement was interpreted as follows: k of 0.01–0.20: Slight agreement; k of 0.21–0.40: Fair agreement; k of 0.41–0.60: Moderate agreement; k of 0.61–0.80: Good agreement; and k of 0.80–1.00: Excellent agreement [21]. The diagnostic performance of the treatment response of TACE per tumor, based on three CBCT images after chemoembolization, was evaluated for each reviewer. Multiple logistic regression analysis, which was adjusted by age, sex, and tumor size was performed using C statistics, and odds ratios (ORs) with 95% confidence interval (CIs) were calculated to evaluate the ability to determine treatment response per tumor according to imaging type. PBV_mean_ and PBV_max_ of the liver, and each tumor for pre- and post-chemoembolization were compared using the paired *t*-test. Receiver operating characteristic (ROC) curve analysis was performed to determine a cutoff point of perfusion for predicting treatment response after chemoembolization. The treatment result was evaluated for each tumor based on CBCT-based-liver-perfusion-mapping during TACE, for each operator. The sensitivity, specificity, positive-predictive-value (PPV), negative-predictive-value (NPV), and false-positive-rate (FPR) were calculated to estimate the diagnostic performance of the operator’s judgment. A *p* value < 0.05 was considered to be statistically significant. Statistical analysis was performed using SAS software package (ver. 9.4; SAS Institute, Cary, NC, USA).

## 3. Results

### 3.1. Patient Demographics and Treatment Response

**Figure 2:**
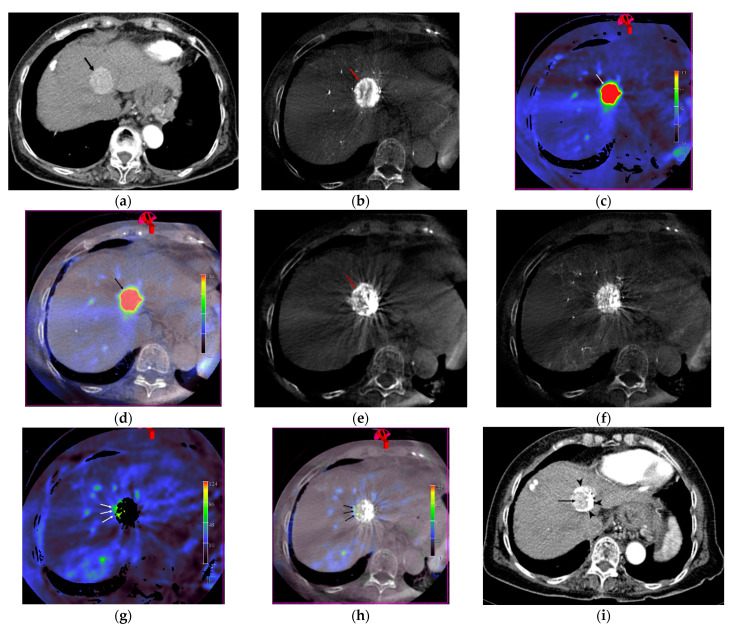
A 72-year-old woman with advanced liver cirrhosis and hepatocellular carcinoma (HCC) in segment 4 of the liver. She underwent transarterial chemoembolization. (**a**) An approximately 3.0 × 2.6-cm enhancing mass (arrow) is noted in segment 4 of the liver in the arterial phase of contrast-enhanced dynamic computed tomography (CT) of the liver. (**b**) A contrast-enhancing mass (arrow) is demonstrated on enhanced cone-beam CT. (**c**,**d**) The mass (arrow in **c**, **d**) shows increased perfusion, seen in red, on cone-beam CT-based perfusion-mapping; pure perfusion imaging (**c**) and fusion image of perfusion image and pre-transarterial chemoembolization (TACE) unenhanced CBCT image (**d**). (**e**) Compact ethiodized oil deposition is noted at the previously noted viable tumor (arrow) on unenhanced cone-beam CT performing after chemoembolization. (**f**) A viable tumor is not clearly observed on enhanced cone-beam CT performing after chemoembolization. (**g**,**h**) A focal, nodular, increased perfusion area, seen in yellow and light green color (arrow in **g**, **h**), suspicious for residual tumor, is demonstrated at the medial aspect of the mass by ethiodized oil deposition on cone-beam CT-based liver-perfusion-mapping performed after chemoembolization; pure perfusion imaging (**g**) and fusion image of perfusion image and post-TACE unenhanced cone beam computed tomography (CBCT) image (**h**). (**i**) CT image obtained 3 months after transarterial chemoembolization shows an enhancing portion (arrow) at the medial aspect of the treated HCC with ethiodized oil deposition (arrowhead). This enhancing portion (arrow) was presumed to be a viable tumor portion.

Baseline demographics and clinical characteristics of patients are summarized in Table 1. All 35 patients underwent TACE successfully, with complete acquisition of L-CBCT, CE-CBCT, and CBCT-based-liver-perfusion-mapping images, on both pre- and post-chemoembolization. Fifty-nine tumors were treated in the 35 patients, with a mean tumor size of 2.4 cm (range 0.9–15.7 cm). Immediate treatment response was evaluated based on three types of CBCT images (Figure 2). The response outcome in 58 tumors of 34 patients, according to mRECIST, were complete response for 33, partial response for 19, stable disease for 5, and progressive disease for 1. One patient, with a single tumor, underwent liver transplantation after TACE, with histopathological confirmation of a small viable tumor in the extracted liver. Correlation between the treatment response to TACE on three types of CBCT images obtained during the procedure, as determined by each reviewer, and the response outcomes, based on follow-up examination, are summarized in Table 2.

### 3.2. Diagnostic Performance of Treatment Response According to Imaging Type

K statistics for the reviewer’ scores for prediction of treatment response based on the CBCT images were 0.2293 for L-CBCT, 0.0762 for CE-CBCT, and 0.7242 for CBCT-based-liver perfusion-mapping per tumor; good for CBCT-based-liver-perfusion-mapping, fair for L-CBCT, and slight for CE-CBCT in agreement among the three reviewers for prediction of treatment responses. 

In terms of the performance of the three types of images in predicting treatment response, the sensitivity for detecting a viable tumor was lower for L-CBCT and CE-CBCT in all three reviewers. The specificity for detecting viable tumor was high for both L-CBCT and CE-CBCT for reviewers 1 and 2. Sensitivity, specificity, and the overall diagnostic accuracy were higher for CBCT-based-liver-perfusion-mapping, for all three reviewers (Table 3). In terms of the performance of the three types of images in predicting treatment response, only for complete response cases and partial response cases, negative predictive value for detecting a viable tumor was superior for CBCT-based-liver perfusion-mapping in all three reviewers. The positive predictive value for detecting viable tumor was high for both L-CBCT and CE-CBCT for reviewers 1 and 2. There was no significant difference between modalities in sensitivity, specificity, and accuracy in all three reviewers (Table 4).

### 3.3. Ability to Predict Treatment Response According to Imaging Type and Perfusion Parameters

Multiple logistic regression analysis was performed to assess the ability of each imaging type to determine treatment response, after adjustment for age, sex, and tumor size. CBCT-based-liver-perfusion-mapping showed statistically significant better ability to predict treatment response, with the highest C statistic (0.954). The PBV_max_ of the tumor was a statistically significant predictor of treatment response with a C statistic of 0.7844. L-CBCT and CE-CBCT, the PBV_mean_ of the tumor and liver, and the PBV_max_ of liver were not statistically significant predictors of treatment response (Table 5).

### 3.4. Quantitative Analysis of Perfusion Parameters

In paired *t*-test analyses, the PBV_mean_ and PBV_max_ of the tumor, and the PBV_mean_ of the liver were significantly decreased after TACE (each *p* < 0.001). PBV_max_ of the liver was slightly different before vs. after TACE (*p* = 0.0002) (Table 6).

**Figure 3:**
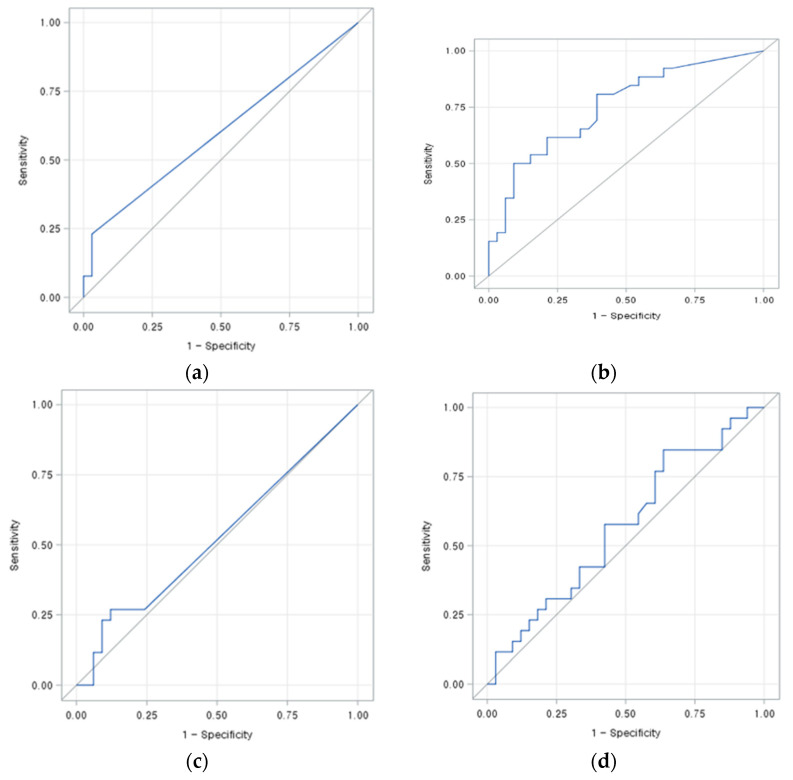
Receiver operating characteristic curve analysis of perfusion parameters of the tumor and liver on cone-beam CT-based perfusion mapping performed immediately after chemoembolization. (**a**) Mean parenchymal blood volume (PBV_mean_) of the tumor: Area under the curve, 0.5991; cutoff value, 0; sensitivity, 23.1%; specificity 97.0%. (**b**) Maximum parenchymal blood volume (PBV_mean_) of the tumor: area under the curve, 0.7523; cutoff value, 19.00; sensitivity, 80.8%; specificity, 60.6%. (**c**) Mean parenchymal blood volume (PBV_mean_) of the liver: Area under the curve, 0.5239; cutoff value, 15.33; sensitivity, 26.9%; specificity, 87.9% (**d**) Maximum parenchymal blood volume (PBV_mean_) of the liver: area under the curve, 0.5682; cutoff value, 79.00; sensitivity, 84.6%; specificity, 36.4%.

In ROC curve analyses (Figure 3), area under curve (AUC) for prediction of residual tumor based on PBV_max_ of the tumor after TACE was 0.7523, using a cutoff value of 19 mL/L, with 80.8% sensitivity and 60.6% specificity. The AUCs of other perfusion parameters, such as PBV_mean_ of the tumor and liver, and PBV_max_ of the liver, were less than 0.6.

### 3.5. Diagnostic Performance of the Operator’s Judgment

The operator’s judgment about the technical results of TACE, according to CBCT-based-liver perfusion-imaging after chemoembolization, yielded a sensitivity of 66.67% (95%CI 48.89–84.45%), specificity of 90.63% (95%CI 80.53–100%), positive-predictive value of 85.71% (95%CI 70.75–100%), negative-predictive value of 76.32% (95%CI 62.80–89.83%), and accuracy of 79.66% (95%CI 69.39–89.93).

## 4. Discussion

In this study, we validated the usefulness of CBCT-based-liver-perfusion-mapping, performed during conventional TACE for assessing the quantitative and qualitative response of HCC to the treatment, as an outcome predictor. The diagnostic performance of CBCT for detection of viable tumor and evaluation of treatment response has been well-studied and its usefulness proven, as compared with that of conventional angiography [13,23]. However, the utility of CBCT-based-liver-perfusion-imaging in evaluation of TACE has not been established.

In our previous retrospective study, conventional angiography fared far worse in qualitative evaluation than the other three types of CBCT images [16]. Therefore, we excluded conventional angiography from this study, and only compared unenhanced CBCT, enhanced CBCT, and CBCT-based-liver-perfusion-imaging, obtained during TACE, to evaluate their capability for viable tumor detection and prediction of treatment response. The present prospective and previous retrospective study differ in a number of respects. First, the diagnostic performance for evaluating treatment response according to image type differed in these studies. According to our prior study, CBCT-based-liver-perfusion-imaging was superior in overall diagnostic performance, but comparable to unenhanced cone-beam CT. However, in this study, CBCT-based-perfusion-imaging showed greater sensitivity and accuracy for detection of viable tumor after chemoembolization than both unenhanced and enhanced CBCT. The second difference relates to treatment response. Although, the prior study showed that PBV_mean_ of the tumor and CBCT-based-liver-perfusion-imaging were significant predictors of treatment response; unenhanced CBCT and enhanced CBCT were also statistically comparable, with a minor difference. However, in the present study, only CBCT-based-liver-perfusion-imaging and PBV_max_ of the tumor were statistically significant predictors of treatment response. These different results between the two studies are thought to be due to limitations of unenhanced- and enhanced CBCT. In unenhanced CBCT, ethiodized oil deposition is the only evaluation parameter for determining whether there are viable portions or not. In enhanced CBCT, it is difficult to distinguish a small enhancing portion from ethiodized oil staining in HCC. Consequently, small viable portions of HCCs are often underestimated or overestimated during TACE, resulting in low sensitivity and accuracy for detection of viable portions in unenhanced and enhanced CBCT. We considered that these factors may have biased the reading of unenhanced- and enhanced CBCT, which might have made a difference according to individual situation in the two studies. Thirdly, in the prior retrospective study, two reviewers were abdominal radiologists, and in this study, all three reviewers were interventionist. Therefore, there may be a difference in interpreting the unenhanced-and enhanced CBCT. However, CBCT-based-perfusion-imaging allows intuitive analysis of images by color, with high confidence for detecting viable tumor, perfusion images were superior in both the prior retrospective and this prospective study.

We also assessed the ability of the operator’s judgment about the presence or absence of a viable remaining tumor portion in treated HCC during the procedure to predict treatment response. We found that the operator’s judgement of the technical results during TACE, according to CBCT-based-perfusion-imaging, was superior to the three reviewer’s analysis of unenhanced and enhanced CBCT images. Therefore, CBCT-based-perfusion-imaging may facilitate achieving successful treatment results and may positively affect TACE. The diagnostic performance of the operator’s judgement was slightly lower than that of the three reviewers’ analyses of CBCT-based-perfusion-imaging, which may be due to the operator’s subjectivity during the procedure.

This study had some limitations. Although it was a prospective study, it was a single-center study with a small number of patients, which may not be addressed until CBCT-based-perfusion-mapping becomes more widespread. Numerous simple steps can be taken to reduce radiation exposure, to reduce the scan time to improve patient cooperation, particularly in terms of breath-holding, and to ensure objective interpretation of CBCT-based-perfusion-imaging to facilitate clinical implementation of this modality. Also, this study is limited to conventional TACE with ethiodized oil, and it is not known whether it will be effective in drug-eluting bead TACE. Since ethiodized oil is easily confused with iodine contrast agents, the perfusion image expressed in color is thought to have been remarkable in the conventional TACE with ethiodized oil. We believe that the advantages of perfusion image are relatively less likely to be highlighted because there are no disadvantages of such ethiodized oil in drug-eluting bead TACE. However, in any situation, images expressed in color appear more intuitive than black-and-white images, so we expect the advantage of perfusion images to still emerge in drag-eluting bead TACE.

## 5. Conclusions

CBCT-based-liver-perfusion-mapping, performed immediately after TACE for HCC, was useful for assessing response to TACE, both quantitatively and qualitatively, and for predicting the response outcome.

## Figures and Tables

**Table 1:** Baseline demographics and clinical characteristics of patients.

Clinical Characteristics		Value
Sex	Man	31
	Woman	4
Age, median (range)(year)		66 (45–85)
Cause of liver cirrhosis	HBV	20
	HCV	4
	Alcohol	4
	HBV + alcohol	2
	NBNC	5
Child-turotte-pugh class	A	29
	B	6
ECOG performance status	0	33
	1	2
BCLC stage	0	10
	A	19
	B	6
Previous treatment of HCC	No treatment	15
	RFA	1
	TACE	18
	TACE + RFA	1
Tumor multiplicity	Single	21
	Multiple, two nodules	5
	three nodules	8
	four nodules	1
HCC diagnosis modality	CT	17
	MRI	18
Follow-up examination	CT	23
	MRI	10
	Pathology	1

HBV: hepatitis B virsu; HCV: hepatitis C virus; NBNC: non-B, non-C: ECOG: Eastern Cooperative Oncology Group; BCLC: Barcelona Clinic Liver Cancer; HCC: hepatocellular carcinoma; RFA: Radiofrequency ablation; TACE: transarterial chemoembolization; CT: computed tomography; MRI: magnetic resonance imaging.

**Table 2:** Correlation between treatment response on imaging modalities after transarterial chemoembolization for hepatocellular carcinoma and response outcomes on follow-up CT or MRI.

Reviewer	Modality	TACE Result (Treatment Response)	* Response Outcome (*n* = 59)			
			CR	PR	SD	PD
R1	L-CBCT	0 (S)	19	10	3	1
		1 (S)	10	6	2	0
		2 (F)	3	5	0	0
		3 (F)	0	0	0	0
	CE-CBCT	0 (S)	11	6	0	0
		1 (S)	17	12	4	0
		2 (F)	4	3	1	1
		3 (F)	0	0	0	0
	CBCT-based-liver-perfusion-mapping	0 (S)	11	6	0	0
		1 (S)	7	6	0	0
		2 (F)	9	4	5	0
		3 (F)	5	5	0	1
R2	L-CBCT	0 (S)	8	6	1	0
		1 (S)	21	13	4	1
		2 (F)	3	2	0	0
		3 (F)	0	0	0	0
	CE-CBCT	0 (S)	7	5	0	0
		1 (S)	22	13	5	0
		2 (F)	3	3	0	1
		3 (F)	0	0	0	0
	CBCT-based-liver-perfusion-mapping	0 (S)	6	6	0	0
		1 (S)	17	8	0	0
		2 (F)	4	2	5	0
		3 (F)	5	5	0	1
R3	L-CBCT	0 (S)	5	3	1	1
		1 (S)	13	8	3	0
		2 (F)	12	9	1	0
		3 (F)	2	1	0	0
	CE-CBCT	0 (S)	4	4	1	0
		1 (S)	12	7	1	1
		2 (F)	13	7	3	0
		3 (F)	3	3	0	0
	CBCT-based-liver-perfusion-mapping	0 (S)	9	8	1	0
		1 (S)	11	4	0	0
		2 (F)	4	4	3	0
		3 (F)	8	5	1	1

CT: computed tomography; MRI: magnetic resonance imaging; CR, complete response; PR, partial response; SD, stable disease; PD, progress disease; S, technical success; F, technical failure. L-CBCT: CBCT image on the mask run; Ce-CBCT: CBCT images on the fill run; CBCT: cone-beam computed tomography. * Response outcome was evaluated based on the modified Response Evaluation Criteria in Solid Tumors according to the Society of Interventional Radiology standardization of terminology and reporting criteria [22].

**Table 3:** Diagnostic performance of treatment response evaluation on different imaging modalities after transarterial chemoembolization for hepatocellular carcinoma.

Modality	Reviewer	Sensitivity (95%CI)	Specificity (95%CI)	PPV (95%CI)	NPV (95%CI)	Accuracy (95%CI)
L-CBCT	R1	25.93 (9.4–42.46)	96.88 (90.86–100)	87.5 (64.58–100)	60.78 (47.38–74.18)	64.41 (52.19–76.62)
	R2	14.81 (1.41–28.21)	96.88 (90.86–100)	80 (44.94–100)	57.41 (44.22–70.6)	59.32 (46.79–71.86)
	R3	44.44 (25.7–63.19)	59.38 (42.36–76.39)	48 (28.42–67.58)	55.88 (39.19–72.57)	52.54 (39.8–65.28)
CE-CBCT	R1	25.93 (9.4–42.46)	93.75 (85.36–100)	77.78 (50.62–100)	60 (46.42–73.58)	62.71 (50.37–75.05)
	R2	25.93 (6.72–42.46)	100 (100–100)	100 (100–100)	61.54 (48.32–74.76)	66.1 (54.02–78.18)
	R3	55.56 (36.82–74.3)	56.25 (39.06–73.44)	51.72 (33.53–69.91)	60 (42.47–77.53)	55.93 (43.26–68.6)
CBCT-based-liver-perfusion mapping	R1	96.3 (89.18–100)	90.63 (80.53–100)	89.66 (78.58–100)	96.67 (90.25–100)	93.22 (86.81–99.64)
	R2	81.48 (47.43–96.13)	100 (100–100)	100 (100–100)	86.49 (75.48–97.5)	91.53 (84.42–98.63)
	R3	85.19 (71.79–98.59)	90.63 (80.53–100)	88.46 (76.18–100)	87.88 (76.74–99.02)	88.14 (79.88–96.39)

CI, confidence interval; PPV, positive-predictive values; NPV, negative-predictive values. L-CBCT: CBCT image on the mask run; Ce-CBCT: CBCT images on the fill run; CBCT: cone-beam computed tomography.

**Table 4:** Diagnostic performance of treatment response evaluation on different imaging modalities after transarterial chemoembolization for hepatocellular carcinoma for complete response and partial response.

Modality	Reviewer	Sensitivity (95%CI)	Specificity (95%CI)	PPV (95%CI)	NPV (95%CI)	Accuracy (95%CI)
L-CBCT	R1	64.44 (48.78–78.13)	62.5 (24.49–91.48)	90.62 (74.98–98.02)	23.81 (8.22–47.17)	64.15 (49.8–76.86)
	R2	60.42 (45.27–74.23)	40 (5.27–85.34)	90.62 (74.98–98.02)	9.52 (1.17–30.38)	58.49 (44.13–71.86)
	R3	62.07 (42.26–79.31)	41.67 (22.11–63.36)	56.25 (37.66–73.64)	47.62 (25.71–70.22)	52.83 (38.64–66.7)
CE-CBCT	R1	58.7 (43.23–73)	42.86 (9.9–81.59)	87.1 (70.17–96.37)	13.64 (2.91–34.91)	56.6 (42.28–70.16)
	R2	61.7 (46.38–75.49)	50 (11.81–88.19)	90.62 (74.98–98.02)	14.29 (3.05–36.34)	60.38 (46–73.55)
	R3	59.26 (38.8–77.61)	38.46 (20.23–59.43)	50 (31.89–68.11)	47.62 (25.71–70.22)	49.06 (35.06–63.16)
CBCT-based liver-perfusion mapping	R1	60 (40.6–77.34)	39.13 (19.71–61.46)	56.25 (37.66–73.64)	42.86 (21.82–65.98)	50.94 (36.84–64.94)
	R2	62.16 (44.76–77.54)	43.75 (19.75–70.12)	71.88 (53.25–86.25)	33.33 (14.59–56.97)	56.6 (42.28–70.16)
	R3	62.5 (43.69–78.9)	42.86 (21.82–65.98)	62.5 (43.69–78.9)	42.86 (21.82–65.98)	54.72 (40.45–68.44)

CI, confidence interval; PPV, positive-predictive values; NPV, negative-predictive values; L-CBCT: CBCT image on the mask run; Ce-CBCT: CBCT images on the fill run; CBCT: cone-beam computed tomography.

**Table 5:** Prediction of treatment response according to imaging type and perfusion parameters.

Model		Odds Ratio (95% CI)	*p* Value	C-Statistics
1	Unenhanced cone beam CT	1.221 (0.389–3.835)	0.7323	0.726
2	Contreast-enhanced cone-beam CT	1.632 (0.516–5.161)	0.4047	0.73
3	Cone-beam CT-based-liver-perfusion-mapping	72.661 (10.257–514.756)	<0.0001	0.954
4	PBVmean of tumor	1.032 (0.984–1.081)	0.1964	0.7593
5	PBVmax of tumor	1.012 (1.000–1.023)	0.0488	0.7844
6	PBVmean of liver	1.014 (0.971–1.060)	0.5253	0.7372
7	PBVmax of liver	1.001 (0.997–1.005)	0.6958	0.7401

CI, confidence interval; PBV_mean_, mean value of parenchymal blood volume; PBV_max_, maximum value of parenchymal blood volume.

**Table 6:** Quantitative analysis of perfusion parameters of the tumor and liver, measured on axial images of cone-beam CT-based perfusion mapping, showing the maximum diameter of the tumor before and after transarterial chemoembolization for hepatocellular carcinoma.

Region of Interest	Perfusion Factor	Before TACE (mL/L)	After TACE (mL/L)	*p* Value
Tumor	PBVmean	113.09 ± 56.42	7.02 ± 26.5	<0.0001
	PBVmax	195.32 ± 84.77	57.27 ± 77.91	<0.0001
Liver	PBVmean	26.04 ± 21.82	6.67 ± 13.69	<0.0001
	PBVmax	273.92 ± 150.35	204.32 ± 140.1	0.0002

PBV_mean_, mean value of parenchymal blood volume; PBV_max_, maximum value of parenchymal blood volume; TACE: transarterial chemoembolization.

## Data Availability

Data sharing is not applicable to this article.
